# Overexpression of *TRPV3* Correlates with Tumor Progression in Non-Small Cell Lung Cancer

**DOI:** 10.3390/ijms17040437

**Published:** 2016-03-24

**Authors:** Xiaolei Li, Qianhui Zhang, Kai Fan, Baiyan Li, Huifeng Li, Hanping Qi, Jing Guo, Yonggang Cao, Hongli Sun

**Affiliations:** 1 Department of Pathology, Harbin Medical University-Daqing, Daqing 163319, China; xiaoleili2004@163.com; 2 Department of Scientific Research, Third Affiliated Hospital of Guizhou Medical University, Duyun 558000, China; 3 Department of Pharmacology, Harbin Medical University-Daqing, Daqing 163319, China; maomaozqh@sina.com (Q.Z.); 18385653360@163.com (H.Q.); songbaili@126.com (J.G.); 4 Department of Pathophysiology, Harbin Medical University-Daqing, Daqing 163319, China; katie8213@sina.com; 5 Department of Pharmacology, Harbin Medical University, Harbin 150081, China; liyingxu2004@126.com; 6 Department of Pathology, Daqing General Hospital Group Oilfield General Hospital, Daqing 163319, China; car_fast_2003@163.com

**Keywords:** non-small cell lung cancer, TRPV3, proliferation, [Ca^2+^]_i_, cell cycle

## Abstract

(1) Background: Transient receptor potential vanilloid 3 (*TRPV3*) is a member of the TRP channels family of Ca^2+^-permeant channels. The proteins of some TRP channels are highly expressed in cancer cells. This study aimed to assess the clinical significance and biological functions of TRPV3 in non-small cell lung cancer (NSCLC); (2) Methods: Immunohistochemistry was used to detect the expression of *TRPV3* in NSCLC tissues and adjacent noncancerous lung tissues. Western blot was used to detect the protein expressions of *TRPV3*, *CaMKII*, *p-CaMKII*, *CyclinA*, *CyclinD*, *CyclinE1*, *CDK2*, *CDK4*, and *P27*. Small interfering RNA was used to deplete *TRPV3* expression. A laser scanning confocal microscope was used to measure intracellular calcium concentration ([Ca^2+^]_i_). Flow cytometry was used to analyze cell cycle; (3) Results: *TRPV3* was overexpressed in 65 of 96 (67.7%) human lung cancer cases and correlated with differentiation (*p* = 0.001) and TNM stage (*p* = 0.004). Importantly, *TRPV3* expression was associated with short overall survival. In addition, blocking or knockdown of *TRPV3* could inhibit lung cancer cell proliferation. Moreover, *TRPV3* inhibition could decrease [Ca^2+^]_i_ of lung cancer cells and arrest cell cycle at the G1/S boundary. Further results revealed that *TRPV3* inhibition decreased expressions of *p-CaMKII*, *CyclinA*, *CyclinD1*, *CyclinE*, and increased *P27* level; (4) Conclusions: Our findings demonstrate that *TRPV3* was overexpressed in NSCLC and correlated with lung cancer progression. *TRPV3* activation could promote proliferation of lung cancer cells. *TRPV3* might serve as a potential companion drug target in NSCLC.

## 1. Introduction

Lung cancer is the world’s leading cause of cancer death [1]. There are two main types of lung cancer: non-small cell lung cancer (NSCLC) and small cell lung cancer (SCLC). The majority of the diagnosed lung cancer cases are NSCLC (80% to 85%) [2]. Although surgical resection, chemotherapy, and radiotherapy have been established, the five-year survival rate for lung cancer patients is still generally poor [3,4,5]. Recent studies propose that conventional treatment may have reached a therapeutic plateau, so the demanding tasks are to elucidate the pathogenesis of lung cancer biology and to explore novel therapeutic targets.

Transient receptor potential (TRP) channels belong to a large family of non-selective cation channels [6,7]. TRP channels have an important effect on cellular functions and signaling pathways as significant mediators of sensory signals [8]. There has been a recent upsurge in the amount of work that expands TRP channel drug discovery efforts into new disease areas, such as anxiety, asthma, cardiac hypertrophy, obesity, as well as cancer and metabolic disorders [9]. Transient receptor potential vanilloid 3 (*TRPV3*) is a member of the TRP channel family of Ca^2+^-permeant channels, which is located on chromosome 17p13 and expressed in skin, tongue, dorsal root ganglion, trigeminal ganglion, spinal cord, and the brain [6]. *TRPV3* function has also been demonstrated in skin barrier formation, hair growth, wound healing, keratinocyte maturation, cutaneous pain, itch, and temperature sensations [10]. Previous reports indicated that the proliferation rate in the oral epithelia of *TRPV3* knockout mice was less than that of wild-type mice [11], and *TRPV3* upregulation was also shown to be associated with a high risk for development of colorectal cancer [12].

The protein expression of *TRPV3* in non-small cell lung cancer and its relationship with clinicopathological factors have not yet been examined. In addition, the biological roles of *TRPV3* in lung cancer cells are still unclear. To solve the problems above, immunohistochemical staining was used to detect the expression of *TRPV3* in non-small-cell lung cancer tissues. Moreover, the effect of *TRPV3* on the proliferation ability of lung cancer cells was also investigated.

## 2. Results

### 2.1. Expression of TRPV3 in Human NSCLCS and Correlation with Clinical Factors

**Figure 1:**
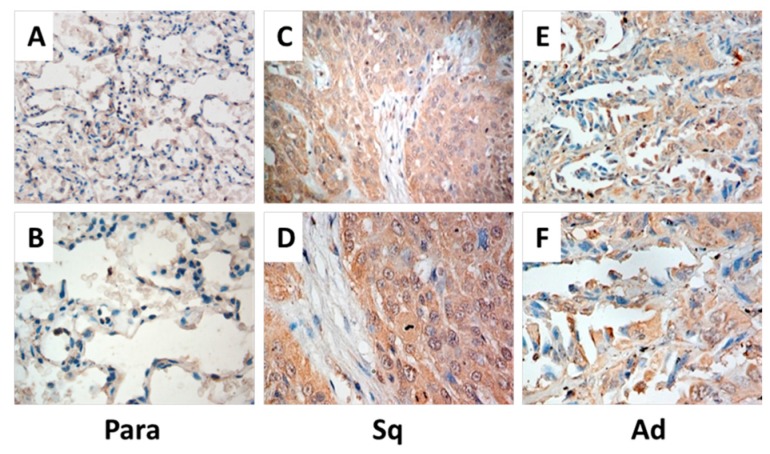

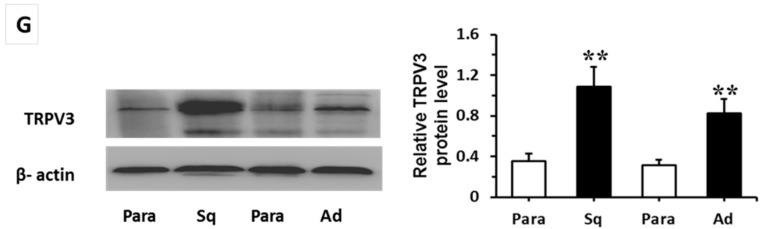
Expression of *TRPV3* in human NSCLCS. Immunohistochemical staining of *TRPV3* was examined in cancerous tissues and their corresponding paracancerous tissues. Brown grains represented positive signals. The positive expression site of *TRPV3* was mainly localized in the cell membrane and cytoplasm of tumor cells. *TRPV3* negative expression was in paracancerous tissue (Para) (**A**,**B**), and *TRPV3* positive expression was in lung squamous cell carcinoma (Sq) (**C**,**D**) and lung adenocarcinoma (Ad) (**E**,**F**). (**A**,**C**,**E**) 200×; and (**B**,**D**,**F**) 400×. The expression of *TRPV3* in lung Ad, lung Sq, and Para (**G**) was determined by Western blot. Each bar represents the mean ± SD of three independent experiments. ** *p* < 0.01 compared with paracancerous tissue.

**Figure 2:**
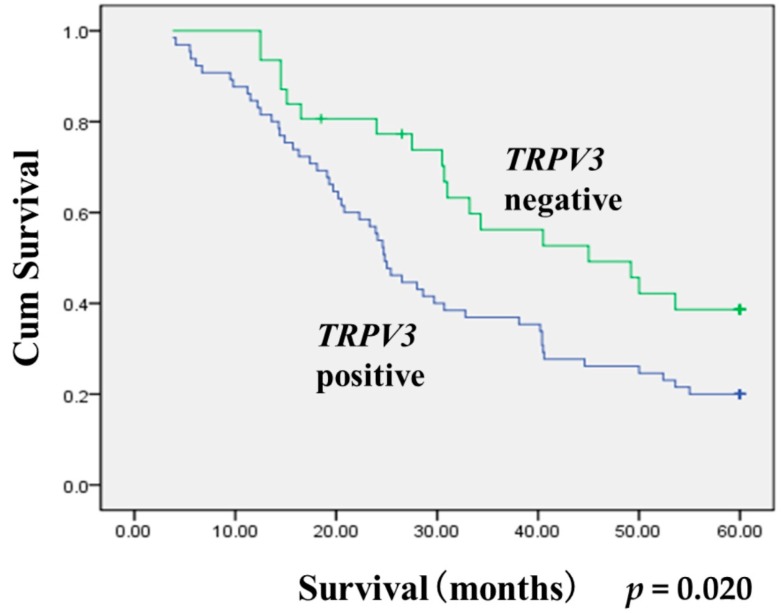
The Kaplan–Meier survival curves. Kaplan–Meier curves for cum survival rates according to *TRPV3* expression status (*p* = 0.020) in NSCLC. Statistical differences were calculated through log-rank comparisons.

We investigated *TRPV3* expression in 96 cases of NSCLC tissue and 20 cases of normal lung tissue by immunohistochemistry. As described above, *TRPV3* immunoreactivity was graded as negative and positive. Positive *TRPV3* staining was observed in 67.7% (65/96) cases; negative *TRPV3* staining was observed in 32.3% (31/96) cases. The *TRPV3* protein was mainly expressed in the cell membrane and cytoplasm (Figure 1A–F). As for the normal lung tissues, only 40% (8/20) of cases had membrane and cytoplasm expression. We analyzed the relationship between *TRPV3* expression levels and clinicopathological factors. As described in Table 1, *TRPV3* expression was significantly correlated with differentiation (*p* = 0.001) and TNM stage (*p* = 0.004). No significant difference in the *TRPV3* status was observed according to the age, gender, smoke, histology, and angiolymphatic invasion. The Western blot analysis results corroborated with the immunohistochemical results. The protein expression of *TRPV3* in NSCLC tissues was significantly higher than that of their paracancerous tissues (Figure 1G). We analyzed the relation of *TRPV3* expression to the overall survival rate and found that the overall survival was significantly lower in patients with *TRPV3* positive NSCLC than in patients with *TRPV3* negative NSCLC (*p* = 0.020 Figure 2).

### 2.2. Activation of TRPV3 Promotes Proliferation of Lung Cancer Cells

**Figure 3:**
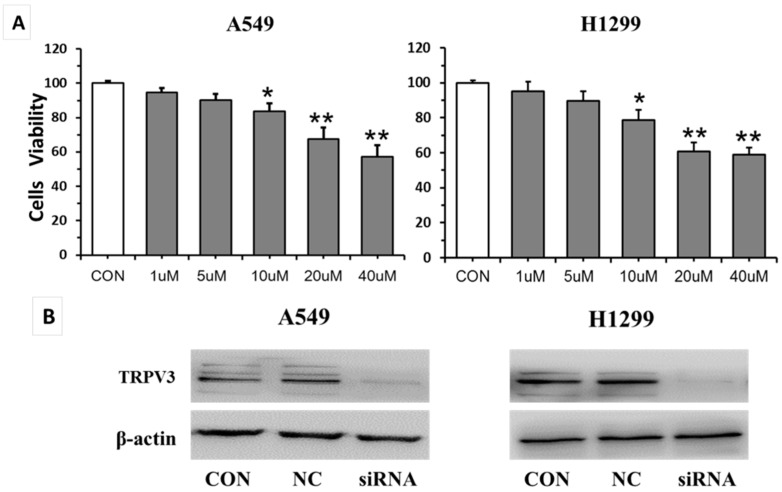

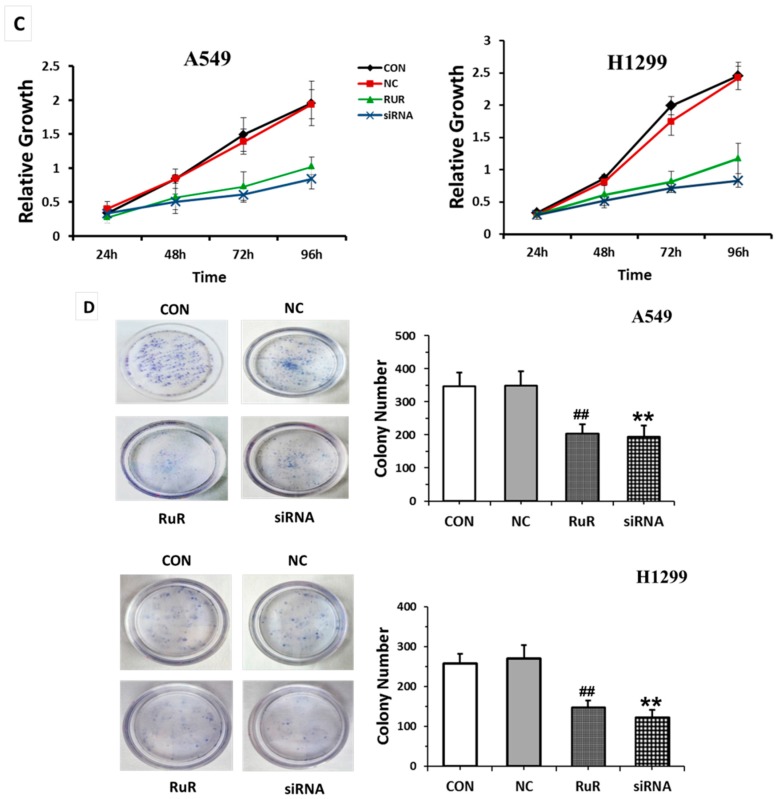
The effect of *TRPV3* on lung cancer cell proliferation. The effect of RuR (1–40 µM) on A549 and H1299 cells after 24 h was analyzed by MTT (**A**); data are expressed as mean ± SD of triplicates. * *p* < 0.05 compared with control group; ** *p* < 0.01 compared with control group. A549 and H1299 cells were treated with *TRPV3*-specific siRNA for 24 h. Western blot of *TRPV3* depletion efficiency in A549 and H1299 cells (**B**); cell growth rate was analyzed in A549 and H1299 cells treated with 20 µM RuR or *TRPV3*-specific siRNA by MTT (**C**) and colony formation assay (**D**). Data are expressed as mean ± SD of triplicates. ^##^ *p* < 0.01 compared with control group; ** *p* < 0.01 compared with NC group.

In order to explore the biological function of *TRPV3* in lung cancer, we used ruthenium red (RuR, a broad spectrum calcium channel blocker) and siRNA techniques to block and knockdown *TRPV3* expression in A549 and H1299 cell lines. The effect of RuR on A549 and H1299 cells was examined using MTT assay (Figure 3A). RuR (1–40 μM) dose-dependently reduced A549 and H1299 cell viability. *TRPV3*-specific siRNA considerably reduced protein expression levels of *TRPV3* after 24 h of siRNA treatment by Western blot assay (Figure 3B). Our cell proliferation analysis showed that the blocking or depletion of *TRPV3* in A549 and H1299 cells led to a significant reduction of the proliferation rate (*p* < 0.05) (Figure 3C). We utilized an independent method, colony formation assays, to validate the anti-proliferative effect of *TRPV3* inhibition in lung cancer cells. The result showed that the blocking or depletion of *TRPV3* in A549 and H1299 cells led to a clear reduction of the colony formation capacity compared to control cells (Figure 3D). These studies demonstrated that the activation of *TRPV3* modulated proliferation of lung cancer cells.

### 2.3. Inhibition of TRPV3 Induces Changes of [Ca^2+^]_i_ in Lung Cancer Cells

**Figure 4:**
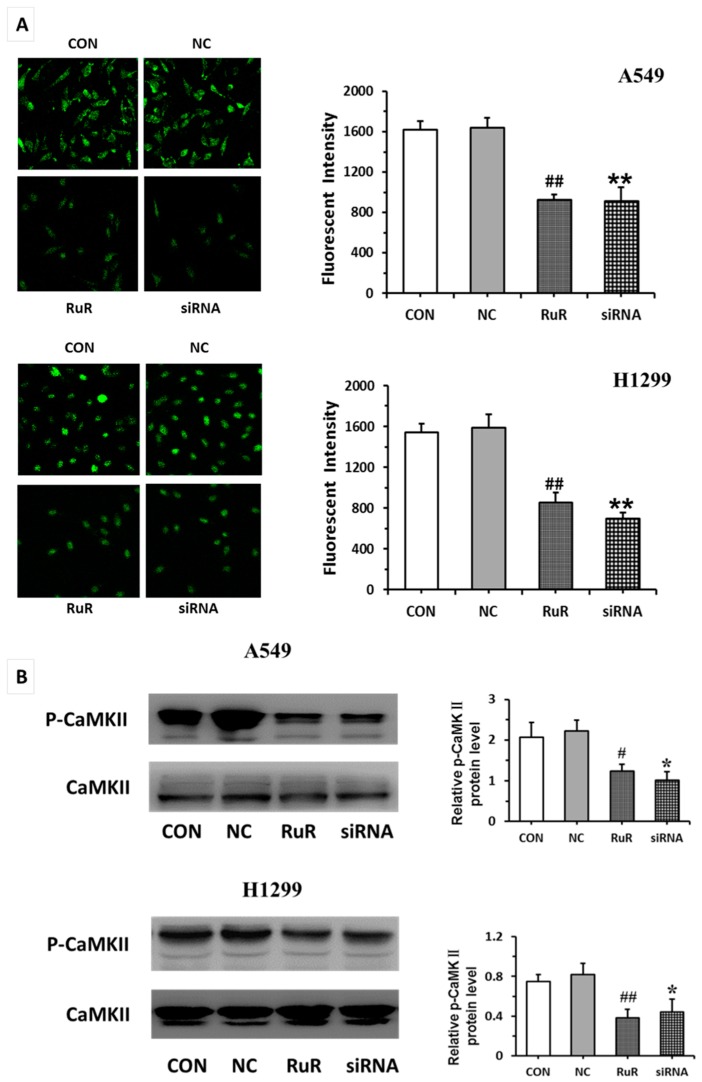
Measurement of [Ca^2+^]_i_ in A549 and H1299 cells. Fluorescent intensity in [Ca^2+^]_i_ was recorded by laser scanning confocal microscope in different treatments (400×) (**A**); Western blot assay for *p-CaMKII* expression in A549 and H1299 cells (**B**). All data were expressed as mean ± SD of triplicates. **^#^** *p* < 0.05 compared with control group; **^##^** *p* < 0.01 compared with control group; * *p* < 0.05 compared with NC group; ** *p* < 0.01 compared with NC group.

From fluorescence intensity taken by laser scanning confocal microscope, we found that blocking or knockdown of *TRPV3* remarkably decreased [Ca^2+^]_i_ of lung cancer cells (Figure 4A). The calcium/calmodulin-dependent kinase II (*CaMKII*) is known to be activated by intracellular Ca^2+^ concentration [13]. Phospho-CaMKII (*p-CaMKII*) protein levels were examined by Western blot. We found that blocking or knockdown TRPV3 decreased expression of *p-CaMKII* (Figure 4B).

### 2.4. Inhibition of TRPV3 Induces Cell Cycle Arrest at the G1/S Boundary in Lung Cancer Cells

**Figure 5:**
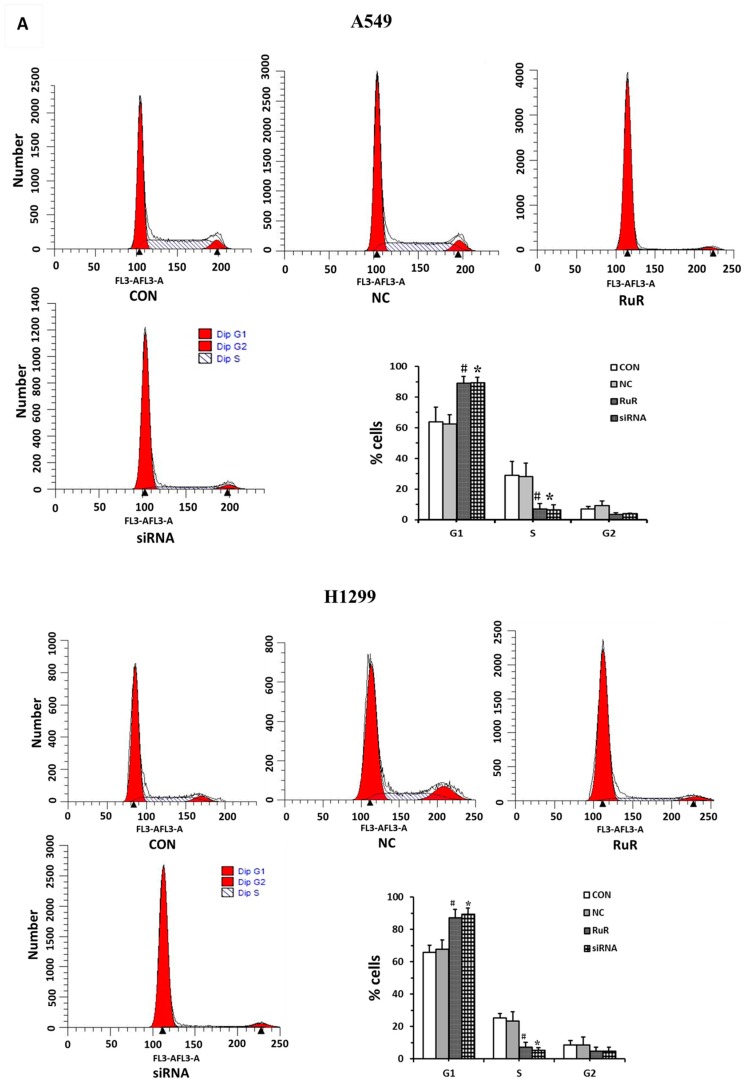

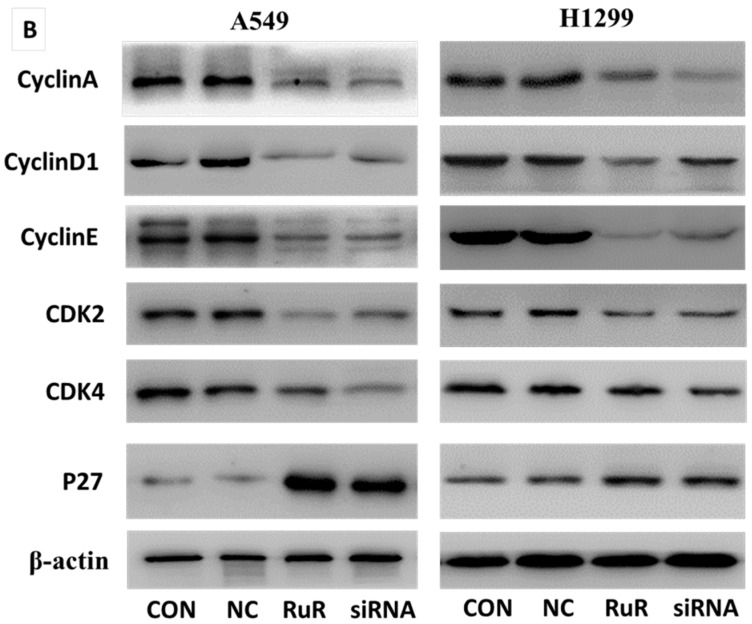
The effects of *TRPV3* on A549 and H1299 cell cycle kinetics. Cell cycle phase was determined from the incorporation of propidium iodide (PI). Cells were analyzed 24 h after plating by flow cytometry (**A**); Western blot analysis of a series of cell cycle related factors showed the protein levels were changed (**B**). All data were expressed as mean ± SD of triplicates. ^#^ *p* < 0.05 compared with control group;* *p* < 0.05 compared with NC group.

To further explore the mechanisms by which *TRPV3* activation promotes NSCLC cell proliferation, the cell cycle analyses were performed in A549 and H1299 cells. Analysis of cell cycle showed that in blocking or in *TRPV3* knockdown cells, the percentage of the S phase was much lower than control cells and the percentage of the G1 phase increased (Figure 5A). These results indicated that *TRPV3* depletion induced cell cycle arrest at the G1/S boundary, and further to inhibit cell cycle progression. To investigate the mechanism underlying cell cycle arrest, we tested the effect of *TRPV3* blocking or knockdown on *CyclinA*, *CyclinD1*, *CyclinE*, *CDK2*, *CDK4*, and *P27* levels. As shown in Figure 5B, Western blot analysis revealed that knockdown of *TRPV3* decreased the protein levels of *CyclinA*, *CyclinD1*, *CyclinE*, and increased *P27* expression. Together, these results suggested that inhibiting *TRPV3* expression induced cell cycle arrest at the G1-S transition and suppressed A549 and H1299 lung cancer cell growth.

## 3. Discussion

In this study, the expression property and biological function of the *TRPV3* protein were detected in human NSCLC. The experimental results showed that the expression of *TRPV3* protein in NSCLC tissues was significantly higher compared with that in the adjacent normal lung tissues. There was a close correlation between *TRPV3* upregulation and TNM stage and differentiation. In addition, we demonstrated that *TRPV3* upregulation also correlated with a shorter survival rate of NSCLC patients.

Studies have demonstrated that some other TRP channels are highly expressed in cancer cells, and the amount of protein expresssion varies with the progression from normal to tumorigenic to metastatic cells, such as *TRPM1*, *TRPM8*, and *TRPV6* [14,15,16,17]. *TRPV1*, *TRPC1*, *TRPC6*, and *TRPM5* are also increased in cancer tissues, but further experiments are required to study the underlying mechanisms [18,19,20]. To validate the potential role of *TRPV3* in lung cancer development, we employed TRPV channel blocker RuR and siRNA to inhibit or knockdown *TRPV3* expression in A549 and H1299 cell lines. We found an impaired proliferation capacity and colony formation ability in A549 and H1299 cells after *TRPV3* blocking or knockdown. These results suggested that *TRPV3* expression was significantly correlated with lung cancer cell proliferation. High *TRPV3* protein expression could promote the proliferation of lung cancer cells.

The roles of TRP channels in cancer progression may involve changes in intracellular Ca^2+^. *TRPV3* is a member of the TRP family and we found that blocking or knockdown *TRPV3* remarkably decreased [Ca^2+^]_i_ of A549 and H1299 lung cancer cells by laser scanning confocal microscope. *CaMKII*, as a general integrator of Ca^2+^ signaling, is activated upon binding of Ca^2+^/calmodulin (*CaM*), which undergoes autophosphorylation [21]. As stated previously, *CaMKII* activation is a frequent occurrence in tumors, including ovarian cancer, colon adenocarcinoma, and many tumor cell lines, as many are the factors that modulate the intracellular Ca^2+^ concentration [22,23]. Our results suggested that *TRPV3* activation increased intracellular Ca^2+^ influx, and it also facilitated the phosphorylation of *CaMKII*.

One function of the calcium signal is to promote the initiation of DNA synthesis at the G1/S transition [24]. Recent studies suggested that *CaMKII* played important roles in the control of cell cycle progression and cell proliferation [25,26]. It had been shown that the inhibition of *CaMKII* reduced *CyclinD1* and enhanced the association of *P27* with *CDK2* that resulted in a G1 phase arrest in fibroblasts [27]. Thus, we employed cell cycle analysis and found that *TRPV3* blocking or knockdown cells showed higher levels of the G1 phase and lower levels of the S phase than the control cells. *TRPV3* blocking or knockdown inhibited G1 to S transition in cell cycle progression, which might explain the mechanism of *TRPV3* on lung cancer cell proliferation. To find out the potential mechanism of *TRPV3* on cell cycle regulation, we examined the effect of *TRPV3* blocking or knockdown on a number of G1 to S transition related molecules. We analyzed the levels of *CyclinA*, *CyclinD1*, *CyclinE*, *CDK2*, *CDK4*, and *P27*. We found that the levels of *CyclinA*, *CyclinD1*, and *CyclinE* were decreased after *TRPV3* blocking or knockdown, while the level of *P27* expression was elevated. *CyclinA* is required for cells to progress through the S phase [28]. *CyclinD1* was overexpressed in many kinds of cancers and correlated with cancer cell proliferation. It influenced cell proliferation by regulating cell cycle progression through the G1/S restriction point [29,30,31]. *P27* was called tumor suppressor protein, which contributed to inhibiting kinase activity and blocking the cell cycle progression through G1 to S phase [32,33]. These data showed that *TRPV3* played an important role in cell cycle control of A549 and H1299 lung cancer cells.

Accumulating evidence suggested that the development of some cancers involved ion channel aberrations, notably in the prostate, in which several non-voltage dependent cationic channels of the TRP family were shown to be key players in calcium homeostasis [34]. For instance, *TRPV2*, *TRPV6*, and *TRPM8* were differentially expressed between normal prostate and prostate carcinomas [35,36,37]. Some authors argued that TRP channels were involved in the proliferation of cancer cells, as well as in their resistance to chemotherapeutic agents [38,39,40]. Our data suggested that high *TRPV3* protein expression could promote the proliferation of A549 and H1299 lung cancer cells. However, the mechanism of *TRPV3* on proliferation of lung cancer cells should be addressed in future studies.

In conclusion, the data demonstrated that *TRPV3* expression was associated with NSCLC progression. High *TRPV3* protein expression could promote the proliferation of lung cancer cells. *TRPV3* inhibition decreased [Ca^2+^]_i_ of lung cancer cells and cell cycle arrest at the G1/S boundary. Accordingl, *TRPV3* expression a prognostic marker and, as a non-selective cation channel, a promising target for new therapeutic strategies to treat advanced NSCLC.

## 4. Materials and Methods

### 4.1. Patient Populations and Clinical Specimens

The Ethics Committee of the Harbin Medical University approved this study. Ninety-six NSCLC samples and the corresponding normal lung tissues were obtained from the Daqing General Hospital Group Oilfield General Hospital during 2005–2008. Patients had not been treated with chemotherapy or radiotherapy before the surgery. Tissues were fixed in formalin and embedded in paraffin. Histological types and differentiation levels were categorized referring to the 2004 WHO classification standards, and TNM (T, the size of tumor; N, regional lymph node; M, metastasis) were classified on the base of the 2009 UICC TNM classification. Relevant clinical and pathological characteristics (Table 1) were collected from the patients’ files and/or by telephone surveys with the patient or their family members.

### 4.2. Immunohistochemistry (IHC) and Scoring

The paraffin-embedded tissues were cut into 5 μm sections, placed on slides, and baked at 70 °C for 2 h. The sections were deparaffinized with xylenes and rehydrated. They were subjected to heat-induced antigen retrieval using citrate buffer (10 mM, pH 6.0) in a microwave oven for 8 min and then cooled to room temperature. The sections were incubated with 3% hydrogen peroxide in methanol to remove the endogenous peroxidase activity and then treated with normal serum to block non-specific binding. The slides were then incubated with an anti-*TRPV3* antibody (1:100 Abcam, Cambridge, MA, USA) overnight at 4 °C. As a negative control for the staining procedure, the primary antibody was replaced with PBS. The second antibody was from a SP reagent kit (Zhongshan Biotechnology Company, Beijing, China). After washing, the tissue sections were treated with a biotinylated anti-rabbit second antibody for 15 min, followed by further incubation with streptavidin-horseradish peroxidase complex for 15 min. The sections were stained with diaminobenzidine and then counterstained with hematoxylin. The stained slides were reviewed and scored independently by two pathologists. The proportion (0: none; 1: <25%; 2: 25%–50%; 3: 51%–75%; and 4: >75%) and intensity (0: none; 1: weak; 2: intermediate; and 3: strong) scores were added to obtain a total score, which ranged from 0 to 7. Specimens were categorized into two groups according to their overall scores: (1) negative expression, <4 points; (2) positive expression, 4–7 points.

### 4.3. Cell Culture

Human lung cancer cell lines A549 and H1299 were obtained from the American Type Culture Collection (Manassas, VA, USA). The cells were cultured in DMEM medium (Invitrogen, Carlsbad, CA, USA) containing 10% fetal calf serum (Invitrogen), 100 IU/mL penicillin (Sigma, St. Louis, MO, USA), and 100 μg/mL streptomycin (Sigma).

### 4.4. Western Blot Analysis

Total proteins from the tissues and cell lines were extracted in lysis buffer (Thermo Fisher Scientific, Rockford, IL, USA) and quantified using the Bradford method. Equal amounts of extracted protein (60 μg) were separated using sodium dodecyl sulfate-polyacrylamide gel electrophoresis (SDS-PAGE) and then were transferred onto a polyvinylidene fluoride membrane. The membrane was incubated overnight at 4 °C with the following antibodies-*TRPV3* (1:100, Abcam, Cambridge, MA, USA), *CaMK-II* (1:1000), *p-CaMK-II* (1:1000), *CyclinA* (1:1000), *CyclinD_1_* (1:1000), *CyclinE* (1:1000), *CDK2* (1:1000), *CDK4* (1:1000), *P27* (1:1000) (Cell Signaling Technology, Boston, MA, USA), β-*actin*(1:2000, Santa Cruz Biotechnology, Santa Cruz, CA, USA). After incubation with peroxidase-coupled anti-mouse/rabbit IgG at 37 °C for 2 h, the protein bands were visualized using ECL (Thermo Fisher Scientific) and detected using the BioImaging Systems (UVP Inc., Upland, CA, USA). The relative amounts of protein were calculated with reference to the amount of β-actin protein.

### 4.5. Knockdown of TRPV3 Expression with Small Interfering RNA

Cells (1 × 10^4^) were transfected with X-tremeGENE siRNA transfection reagent (Roche, Penzberg, Germany), applying a combination of two sequence-validated, and knockdown-warranted siRNAs: *TRPV3*-siRNA (20 nM, each, 5′-ACCUGCCUGAUGAAAGCU UTT-3′ and 5′-AAGCUUUCAUAGGCAGGUTT-3′) according to the manufacturer’s instructions (GenePharma Co., Ltd., Shanghai, China). After 96 h of treatment, the cells were split and retransfected with siRNAs to guarantee efficient *TRPV3* knockdown. A commercial GAPDH-siRNA served as a positive control (GenePharma Co., Ltd., Shanghai, China). A green fluorescent protein-tagged, negative control siRNA (NCsiRNA) (GenePharma Co., Ltd., Shanghai, China) was used as a transfection efficiency control and as a negative control for experiments. Proteins were extracted from cells 24 h later and assessed by Western blot.

### 4.6. Cell Viability Assay

Cells were plated in 96-well plates in medium containing 10% FBS at about 2000 cells per well, and the quantitation of cell viability was determined by MTT (3-(4,5-dimethylthiazol-2-yl)-2,5-diphenyltetrazolium bromide) assay. Briefly, 20 μL of 5 mg/mL MTT (Sigma) solution was added to each well and incubated for 4 h at 37 °C, then the media was removed from each well, and the resultant MTT formazan was solubilized in 150 mL DMSO. The results were quantitated spectrophotometrically by using a test wavelength of 490 nm.

### 4.7. Colony Formation Assay

Cells (0.3 × 10^3^) were seeded into 6 cm dishes and maintained in growth medium. After two weeks, cells were fixed with 80% methanol and stained with Giemsa for 15 min. The number of colonies with above 50 cells was recorded. Colonies were photographed and counted.

### 4.8. Cell Cycle Analysis

Cells (500,000) were seeded into 6 cm tissue culture plates and incubated for 24 h. Following treatment with indicated amounts of siRNA or RuR, cells were collected and fixed in 75% ethanol for 24 h, washed with PBS, and stained with 5 mg/mL propidium iodide (PI) in PBS supplemented with RNase A (Roche, Indianapolis, IN, USA) at room temperature for 30 min. DNA content was analyzed by flow cytometry (FACSAriaI, BD Biosciences, Franklin Lakes, NJ, USA).

### 4.9. Measurement of [Ca^2+^]_i_

The cells were incubated with 10 μM Fluo-3/AM (acetoxymethyl ester form, Molecular Probes, Beijing, China) working solution containing 0.03% Pluronic F-127 at 37 °C for 40 min. Subsequently, the cells were washed twice with working solution to eliminate the extracellular Fluo-3/AM. The changes of [Ca^2+^]_i_ were identified by fluorescent intensity (FI). FI of these cells was detected by a laser scanning confocal microscope (Olympus, Tokyo, Japan) with excitation at 488 nm and emission at 530 nm for 5 min. FI was collected in ten randomly chosen cells to calculate the average FI.

### 4.10. Statistical Analysis

SPSS version 16.0 for Windows was used for all analyses. The Student’s *t*-test was used to compare other data. Between these subgroups, positive rates of stain were compared by Chi-square. Survival analysis was performed using Kaplan–Meier curves, and the differences were revealed by log-rank test. *p* < 0.05 was considered statistically significant, and *p* < 0.01 was considered even more strongly.

## Figures and Tables

**Table 1:** Association of *TRPV3* expression in NSCLC with clinical and pathologic factors.

Features	Patients	*TRPV3* Positive (%)	*TRPV3* Negative (%)	*p*
Age (years)				
<60	48	30 (62.5%)	18 (37.5%)	0.275
≥60	48	35 (72.9%)	13 (27.1%)	
Gender				
Male	55	38 (69.1%)	17 (30.9%)	0.737
Female	41	27 (65.9%)	14 (34.1%)	
Smoke				
Yes	49	33 (67.3%)	16 (32.7%)	0.938
No	47	32 (68.1%)	15 (31.9%)	
Histology				
Adenocarcinoma	56	35 (62.5%)	21 (37.5%)	0.197
Squamous Cell	40	30 (75.0%)	10 (25.0%)	
Carcinoma differentiation				
Well	45	23 (51.1%)	22 (48.9%)	0.001 *
Moderate-Poor	51	42 (82.4%)	9 (17.6%)	
TNM stage				
I/II	61	35 (57.4%)	26 (42.6%)	0.004 *
III/IV	35	30 (85.7%)	5 (14.3%)	
Angiolymphatic invasion				
No	54	34 (63.0%)	20 (37.0%)	0.260
Yes	42	31 (73.8%)	11 (26.2%)	

* *p* < 0.05.
